# Supplementary material for: COZOID: contact zone identifier for visual analysis of protein-protein interactions
Source: BMC Bioinformatics. 2018 Apr 6;19:125. doi: 10.1186/s12859-018-2113-6 (PMC5889581; doi:10.1186/s12859-018-2113-6)
Supplement: Supplementary file 2 — Software build. Executable binary file of the software tool. (ZIP 118,784 kb) [file 12859_2018_2113_MOESM2_ESM.zip › cozoid/platform/docs/testng-6.5.1-javadoc.zip!/org/testng/ITestContext.html]

ITestContext (TestNG)


---


|  |  |  |
| --- | --- | --- |
| **Overview** \| **Package** \| **Class** \| **Tree** \| **Deprecated** \| **Index** \| **Help** | |  |
| **PREV CLASS**   **NEXT CLASS** | **FRAMES**    **NO FRAMES**     **All Classes** |  |
| SUMMARY: NESTED \| FIELD \| CONSTR \| METHOD | DETAIL: FIELD \| CONSTR \| METHOD |  |


---


## org.testng Interface ITestContext

**All Superinterfaces:**: IAttributes, java.io.Serializable

**All Known Implementing Classes:**: TestRunner

---

``` public interface ITestContext extends IAttributes ```

This class defines a test context which contains all the information
for a given test run. An instance of this context is passed to the
test listeners so they can query information about their
environment.

**Author:**
:   Cedric Beust, Aug 6, 2004, Alexandru Popescu

---

| **Method Summary** | |
| --- | --- |
| `void` | `addGuiceModule(java.lang.Class<? extends com.google.inject.Module> cls, com.google.inject.Module module)` |
| `void` | `addInjector(java.util.List<com.google.inject.Module> moduleInstances, com.google.inject.Injector injector)` |
| `ITestNGMethod[]` | `getAllTestMethods()` |
| `XmlTest` | `getCurrentXmlTest()` |
| `java.util.Date` | `getEndDate()`             When this test stopped running. |
| `java.lang.String[]` | `getExcludedGroups()` |
| `java.util.Collection<ITestNGMethod>` | `getExcludedMethods()` |
| `IResultMap` | `getFailedButWithinSuccessPercentageTests()` |
| `IResultMap` | `getFailedConfigurations()`             Retrieves information about the failed configuration method invocations. |
| `IResultMap` | `getFailedTests()` |
| `java.util.List<com.google.inject.Module>` | `getGuiceModules(java.lang.Class<? extends com.google.inject.Module> cls)` |
| `java.lang.String` | `getHost()` |
| `java.lang.String[]` | `getIncludedGroups()` |
| `com.google.inject.Injector` | `getInjector(java.util.List<com.google.inject.Module> moduleInstances)` |
| `java.lang.String` | `getName()`             The name of this test. |
| `java.lang.String` | `getOutputDirectory()` |
| `IResultMap` | `getPassedConfigurations()`             Retrieves information about the successful configuration method invocations. |
| `IResultMap` | `getPassedTests()` |
| `IResultMap` | `getSkippedConfigurations()`             Retrieves information about the skipped configuration method invocations. |
| `IResultMap` | `getSkippedTests()` |
| `java.util.Date` | `getStartDate()`             When this test started running. |
| `ISuite` | `getSuite()` |

**Methods inherited from interface org.testng.IAttributes**

`getAttribute, getAttributeNames, removeAttribute, setAttribute`

**Method Detail**

### getName

```
java.lang.String getName()
```

:   The name of this test.

---


### getStartDate

```
java.util.Date getStartDate()
```

:   When this test started running.

---


### getEndDate

```
java.util.Date getEndDate()
```

:   When this test stopped running.

---


### getPassedTests

```
IResultMap getPassedTests()
```

:   **Returns:**: A list of all the tests that run successfully.

---


### getSkippedTests

```
IResultMap getSkippedTests()
```

:   **Returns:**: A list of all the tests that were skipped

---


### getFailedButWithinSuccessPercentageTests

```
IResultMap getFailedButWithinSuccessPercentageTests()
```

:   **Returns:**: A list of all the tests that failed but are being ignored because annotated with a successPercentage.

---


### getFailedTests

```
IResultMap getFailedTests()
```

:   **Returns:**: A map of all the tests that passed, indexed by their ITextMethor. **See Also:**: `ITestNGMethod`

---


### getIncludedGroups

```
java.lang.String[] getIncludedGroups()
```

:   **Returns:**: All the groups that are included for this test run.

---


### getExcludedGroups

```
java.lang.String[] getExcludedGroups()
```

:   **Returns:**: All the groups that are excluded for this test run.

---


### getOutputDirectory

```
java.lang.String getOutputDirectory()
```

:   **Returns:**: Where the reports will be generated.

---


### getSuite

```
ISuite getSuite()
```

:   **Returns:**: The Suite object that was passed to the runner at start-up.

---


### getAllTestMethods

```
ITestNGMethod[] getAllTestMethods()
```

:   **Returns:**: All the test methods that were run.

---


### getHost

```
java.lang.String getHost()
```

:   **Returns:**: The host where this test was run, or null if it was run locally. The returned string has the form: host:port

---


### getExcludedMethods

```
java.util.Collection<ITestNGMethod> getExcludedMethods()
```

:   **Returns:**: All the methods that were not included in this test run.

---


### getPassedConfigurations

```
IResultMap getPassedConfigurations()
```

:   Retrieves information about the successful configuration method invocations.

---


### getSkippedConfigurations

```
IResultMap getSkippedConfigurations()
```

:   Retrieves information about the skipped configuration method invocations.

---


### getFailedConfigurations

```
IResultMap getFailedConfigurations()
```

:   Retrieves information about the failed configuration method invocations.

---


### getCurrentXmlTest

```
XmlTest getCurrentXmlTest()
```

:   **Returns:**: the current XmlTest.

---


### getGuiceModules

```
java.util.List<com.google.inject.Module> getGuiceModules(java.lang.Class<? extends com.google.inject.Module> cls)
```

---


### addGuiceModule

```
void addGuiceModule(java.lang.Class<? extends com.google.inject.Module> cls,
                    com.google.inject.Module module)
```

---


### getInjector

```
com.google.inject.Injector getInjector(java.util.List<com.google.inject.Module> moduleInstances)
```

---


### addInjector

```
void addInjector(java.util.List<com.google.inject.Module> moduleInstances,
                 com.google.inject.Injector injector)
```


---


|  |  |  |
| --- | --- | --- |
| **Overview** \| **Package** \| **Class** \| **Tree** \| **Deprecated** \| **Index** \| **Help** | |  |
| **PREV CLASS**   **NEXT CLASS** | **FRAMES**    **NO FRAMES**     **All Classes** |  |
| SUMMARY: NESTED \| FIELD \| CONSTR \| METHOD | DETAIL: FIELD \| CONSTR \| METHOD |  |


---
